# Supplementary material for: Improved statistical efficiency of simultaneous multi-slice fMRI by reconstruction with spatially adaptive temporal smoothing
Source: Neuroimage. 2019 Dec;203:116165. doi: 10.1016/j.neuroimage.2019.116165 (PMC6854456; doi:10.1016/j.neuroimage.2019.116165)
Supplement: Multimedia component 1 [file mmc1.docx]

# Supporting Information


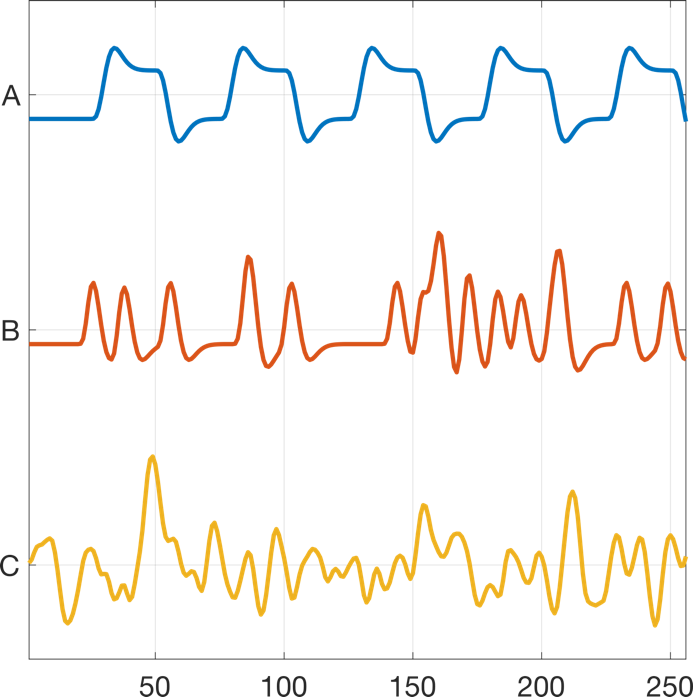


Figure S1 - Example regressors used to assess relative GLM efficiency: (A) 5-period block design task, (B) jittered event-related task, (C) Gaussian white noise regressor. All 3 regressors were convolved with a canonical hemodynamic response function.


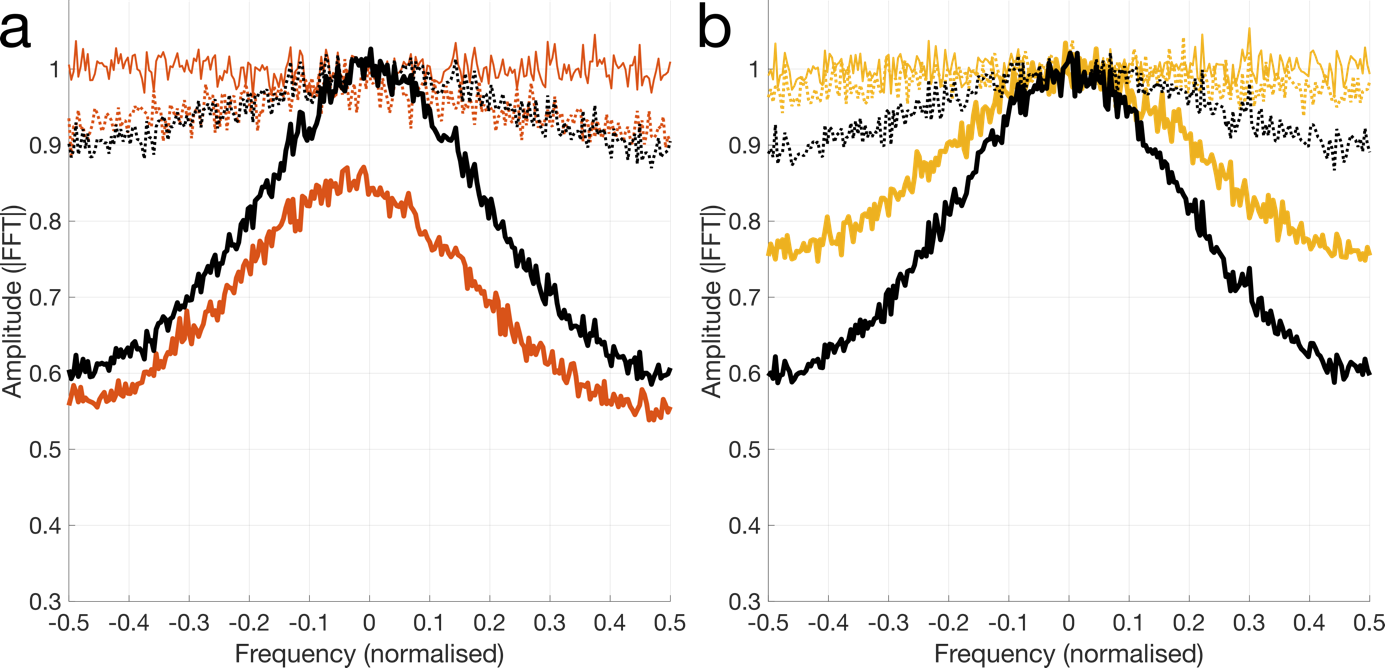


Figure S2 - Noise spectra plotted for additional 2 voxels as a function of of the regularization parameter $\lambda$. Note that the black curves, representing post-hoc kernel smoothing do not change from voxel-to-voxel, and was chosen here to match DOF with the voxel shown in Fig. 2a. Plot color-scheme is matched to those in Fig. 2.


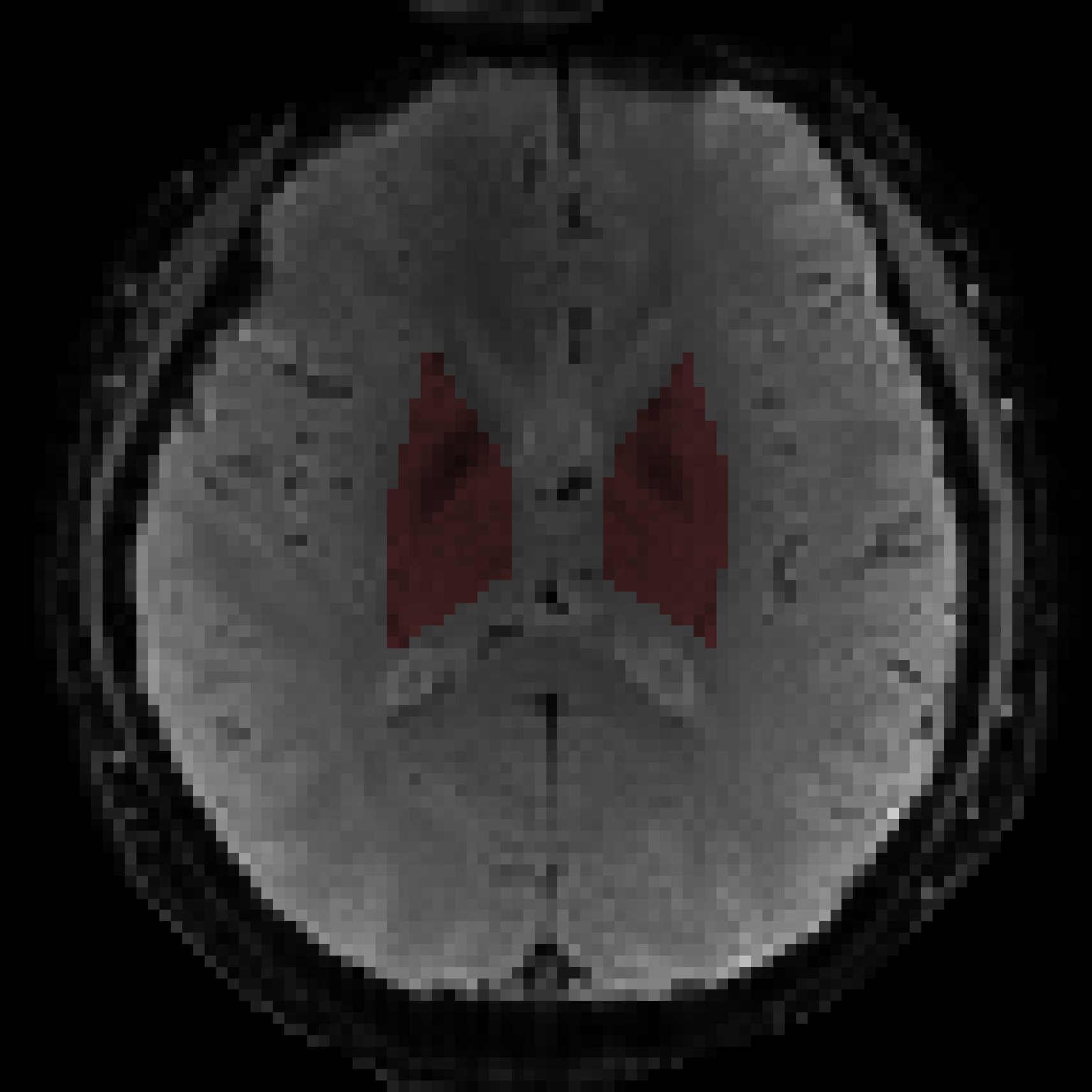


Figure S3 - Deep gray nuceli mask (highlighted in red) including basal ganglia and thalamus, for evaluation of tSNR efficiencies in the MB8R2 data.
